# Intoxicated persons showing challenging behavior demand complexity interventions: a pilot study at the interface of the ER and the complexity intervention unit

**DOI:** 10.1007/s00406-020-01162-7

**Published:** 2020-07-12

**Authors:** Stefan M. H. Verheesen, Freek ten Doesschate, Maarten A. van Schijndel, Rutger Jan van der Gaag, Wiepke Cahn, Jeroen A. van Waarde

**Affiliations:** 1 Department of Psychiatry, Rijnstate Hospital, Arnhem, The Netherlands; 2 Emergency Department, Rijnstate Hospital, Arnhem, The Netherlands; 3 Department of Psychiatry, Radboud University Medical Center, Nijmegen, The Netherlands; 4 Department of Psychosomatics and Psychotherapy, Stradina University, Riga, Latvia; 5 Department of Psychiatry, Utrecht University Medical Center, Utrecht, The Netherlands; 6 Altrecht Science, Altrecht Mental Health Institute, Utrecht, The Netherlands

**Keywords:** Intoxicated persons, Challenging behavior, Emergency room, Integrated healthcare

## Abstract

Intoxicated persons showing challenging behavior (IPCBs) under influence of alcohol and/or drugs frequently have trouble finding appropriate acute care. Often IPCBs are stigmatized being unwilling or unable to accept help. Separated physical and mental healthcare systems hamper integrated acute care for IPCBs. This pilot aimed to substantiate the physical, psychiatric, and social health needs of IPCBs visiting the emergency room (ER) during a 3-month period. All ER visits were screened. After triage by the ER physician, indicated IPCBs were additionally assessed by the consultation–liaison–psychiatry physician. If needed, IPCBs were admitted to a complexity intervention unit for further examinations to provide integrated treatments and appropriate follow-up care. The INTERMED and Health of the Nation Outcome Scale (HoNOS) questionnaires were used to substantiate the complexity and needs. Field-relevant stakeholders were interviewed about this approach for acute integrated care. Alongside substance abuse, almost half of identified IPCBs suffered from comorbid psychiatric disturbances and one third showed substantial physical conditions requiring immediate medical intervention. Almost all IPCBs (96%) accepted the acute medical care voluntarily. IPCBs showed high mean initial scores of INTERMED (27.8 ± 10.0) and HoNOS (20.8 ± 6.9). At discharge from the complexity intervention unit, the mean HoNOS score decreased significantly (13.4 ± 8.6; *P* < 0.001). Field-relevant stakeholders strongly supported the interdisciplinary approach and ER-facility for IPCBs and acknowledged their unmet health needs. A biopsychosocial assessment at the ER, followed by a short admission if necessary, is effective in IPCBs. This approach helps to merge separated healthcare systems and may reduce stigmatization of IPCBs needing help.

## Introduction

Worldwide, intoxicated persons showing challenging behavior (IPCBs) under the influence of alcohol and/or drugs frequently experience trouble finding the most appropriate acute care [1, 2]. In IPCBs, complex interactions exist between physical, psychiatric, and social health (‘biopsychosocial’) problems [3–5]. In some countries, like in the Netherlands, the healthcare system is separated into organizations for physical (e.g., general hospitals) and mental health (e.g., mental healthcare facilities). This hampers the interdisciplinary emergency healthcare that these patients usually require.

### Lack of appropriate care

IPCBs may suffer from acute physical conditions and may present themselves at the emergency room (ER). The number of ER visits by IPCBs is increasing worldwide [6]. ER visits due to consequences of alcohol and/or drugs use are very common (4.9–18%) and causes daily recurring stress for ER professionals [3, 7, 8]. Although not every intoxicated person will have problems with mental health and addiction (e.g., accidentally ingestion of toxic substance), sometimes IPCBs do need above average attention from ER staff because of their simultaneous needs for multiple types of biopsychosocial care. In the USA, for example, 41.3–46.7% of IPCBs showed a comorbid psychiatric disturbance and 29.0–32.8% had injuries related to alcohol intoxication [9]. A negative attitude towards psychiatric patients in the ER is a well-known problem [10, 11], and may hamper appropriate acute care.

Some IPCBs do not reach the ER due to their dangerous or undesirable behavior. Police officers on the streets are increasingly faced with handling these situations. In the Netherlands, a 60% increase of incidents involving persons with challenging behavior was registered by the national police between 2014 and 2017 [12]. As a result, IPCBs are often taken into police custody, even when no severe criminal act has occurred. In the criminal justice system, although, IPCBs may be unmanageable as well due to their medical conditions. In police custody, appropriate medical attention for IPCBs is lacking. Admission of IPCBs directly to the mental healthcare system does, on the other hand, not guarantee adequate physical examination and treatment due to insufficient knowledge about physical problems [13]. Owing to the acute approach, social problems, that cannot be resolved quickly, might be often overlooked (e.g., homelessness, relationship problems, financial problems). An interdisciplinary approach in the emergency healthcare for IPCBs is often missing.

### Stigmatization

Stigmatization seems another major negative factor that hampers appropriate care for IPCBs. There are indications that self-stigma impedes the lives of persons with mental illness by increasing suicidality [14]. The lack of appropriate physical and mental examinations may also be caused by caregivers’ assumptions, for example, that IPCBs are unwilling to accept treatment or simply suffer from social problems only [15]. In daily practice, though, IPCBs often not only suffer from intoxication, but also from (undetected or untreated) psychiatric disorders, intellectual disabilities, and/or underlying substance abuse disorders. However, when IPCBs are in police custody, only a subgroup is medically examined briefly, for example by the mental health crisis intervention team [16]. Subsequently, most IPCBs will not receive appropriate follow-up care when leaving the criminal justice system. Likewise, IPCBs might leave the ER with severe occult conditions if they leave without elaborate physical and psychiatric assessment [17]. Moreover, IPCBs often show a (very) low socio-economic status, especially in high-income countries [18]. When social factors in IPCBs are assessed more extensively, more guidance to appropriate solutions for their problems may be deployed.

### Objective

This pilot aimed to substantiate the physical, psychiatric, and social health needs of IPCBs who would visit the ER during a 3-month period. The main goal was to elucidate and quantify (unidentified and untreated) physical, psychiatric, and/or social crises, and to examine whether a broader biopsychosocial assessment would lead to more appropriate integrated treatment and follow-up care.

We hypothesized that many IPCBs would suffer a diversity of health as well as social problems, of which some would need direct medical interventions. We had some concerns about significant increase in the time to arrange aftercare for complex cases.

## Methods

### Study design

A naturalistic observational cohort study concerning intoxicated patients presenting themselves at the ER of Rijnstate hospital was performed from May 1st until August 1st,  2018. This study concerns a (non-randomized) non-controlled pre–post comparison. Rijnstate is a large teaching hospital in Arnhem, the Netherlands. It has 766 beds and a catchment area of 450,000 citizens. Annually more than 38.000 patients visit the ER of Rijnstate Hospital. The average income in Arnhem is 9.8% lower as compared to the Dutch average income [19].

To improve interdisciplinary triage and acute healthcare, we specifically dedicated the ER for all referred IPCBs during the pilot period. During this pilot period, police officers were allowed to refer IPCBs directly to the ER, after telephone consultation with the psychiatrist on duty to estimate safety risks in advance. If indicated by the ER physician (e.g., ‘confused’ behavior), an additional biopsychosocial assessment was performed at the ER by a consultation–liaison psychiatry physician. If indicated by the consultation–liaison psychiatry physician (e.g., signs of psychiatric symptoms, confused behavior with aggression or self-harm, unclear diagnosis), a consecutive short admittance to the Complexity Intervention Unit followed [20, 21]. See ‘Settings and procedures’ for more details.

In this study, we compared different subgroups to investigate whether there was a difference in severity of illnesses and problems. Furthermore, a qualitative study amongst field-relevant stakeholders was performed to assess the feasibility and quality of the more comprehensive approach. The follow-up period was set to be maximum 48 h unless appropriate follow-up care could not be arranged within these 48 h.

### Subjects

Daily, a psychiatrist screened the list of patients who had been seen at the ER and were designated by the ER physicians and ER nurses with challenging behavior. Persons who were evident or suspected of alcohol and/or drug use and who met one of the following criteria were included: (1) person showed ‘confused’ behavior (assessment made by the referrer); (2) person was not approachable (e.g., not fully conscious, somnolent, sedated); and/or (3) person did not make logical decisions. Because the new approach was meant to be as inclusive as possible, a broad target group was formulated without strict diagnostic criteria (i.e., neither following exactly the classifications of the Diagnostic and Statistical Manual of Mental Diseases nor the International Classification of Diseases). The data were collected and anonymized to fulfill the hospital’s legal obligation to measure and improve quality of healthcare. Therefore, in accordance with Dutch law, informed consent and consultation with a medical ethical committee was not needed. This was confirmed by a formal letter from the local Medical Research Ethics Committee (CMO region Arnhem–Nijmegen).

### Setting and procedures

Patients could be referred through conventional channels (in most cases ambulance staff, general practitioners, mental health institutions, nursery homes, or self-referrals). However, in some incidents, police officers are the first responders to arrive on site. Because of this reason, we also added one new referring channel: the Officer on Duty (Officier van Dienst) from the local police force (Politie Arnhem). After arrival, all subjects were globally assessed by an ER physician and an ER nurse. In case of medical disturbances, other specialists (e.g., surgeon, internist) were consulted by the ER physician. In case of behavioral disturbances, according to the ER-physician’s estimation, the consultation–liaison–psychiatry physician was consulted and a brief biopsychosocial examination in the ER was performed. If there were any medical, psychiatric, or social problems that could benefit from a short admission, the consultation–liaison–psychiatry physician had the opportunity to admit the IPCBs to the complexity intervention unit. At the complexity intervention unit, IPCBs were assessed and treated by a psychiatrist, resident psychiatry and a team of specially trained nurses that are both specialized in medical as well as mental health care. In contradiction to most mental health facilities, our complexity intervention unit is fully medially equipped (e.g., intravenous fluid and medications, oxygen treatment, electrocardiogram, electroencephalogram, and appropriate wound care) and has access to all the hospitals’ facilities (e.g., high-tech laboratory, radiology, direct 24/7 consultation of other medical specialists). Therewith, our facility is specialized in providing integrated assessment and, if necessary, treatments [22]. If it was indicated by the team, further biopsychosocial assessments, indicated treatments, and/or arrangement of appropriate follow-up care were provided.

IPCBs that could be dealt with by the ER-physician alone, or who—after the consultation–liaison–psychiatry physician’s assessment—did not show (severe) biopsychosocial problems, could either be discharged from the ER with a written transfer to the general practitioner or other healthcare provider.

### Quantitative analysis and instruments

To structure further data analyses, each patient’s journey was divided into three clinical processes: (1) initial assessment at the ER; (2) broad biopsychosocial assessment and/or treatments at the ER or complexity intervention unit; and (3) consecutive arrangements for follow-up care after dismissal from the ER or complexity intervention unit. To examine the first process, several patient variables were registered anonymously: sex, age, date and time of arrival, and departure at/from the ER, path of referral, legal context, physical and psychiatric diagnoses, treatments, and interventions provided during admission. To examine the second process, those IPCBs who were more broadly examined by the consultation–liaison–psychiatry physician were additionally rated using the INTERMED questionnaire. This questionnaire is validated to score the complexity of a case by assessing biological, psychological, and social characteristics of hospital patients. The instrument has four main domains in which risk factors concerning history, current state, and prognosis can be scored. A total score > 19 points can be considered as high [23–26]. IPCBs who were subsequently admitted to the complexity intervention unit were additionally rated using the Global Assessment of Functioning (GAF) and the Health of the Nation Outcome Scale (HoNOS) upon arrival and at discharge from the complexity intervention unit; the GAF is a valid measure (range 0–100) of daily functioning, taking into account psychiatric and behavioral disturbance(s) in which lower scores indicate higher disability [27]. The HoNOS is routinely used by mental healthcare services worldwide to map a patient’s mental and social functioning [28, 29]; this instrument is also used as an in-patient comparison to assess efficacy of mental health treatment [30]. All scores from the INTERMED, GAF, and HoNOS were rated by trained and experienced staff (inter-rater reliability of INTERMED: 0.5–0.7 = 0.5–0.7; based on 11 raters’ assessment on three cases with varying complexity). To examine the third process, follow-up arrangements were registered, as well as the date and the time of the first follow-up contact to examine the lead time in the chain care system.

### Statistical analysis

The quantitative results were reported according to the patient’s journey in the three clinical processes. Comparisons were made between IPCBs who were examined by the consultation–liaison–psychiatry physician and those who were not, between IPCBs who were admitted to the complexity intervention unit and those who were not, and between IPCBs for whom police officers were involved at the first moment of the incident and those without police involvement. Based on the type of variable (continuous or dichotomized) and hypothesis tested, parametric tests (Student’s *t* test) or non-parametric tests (Wilcoxon rank sum test or chi-squared test) were used. Statistical analyses were performed in *R* version 3.4.1 (R Core Team, 2017; https://www.R-project.org/) [31]. *P* value < 0.05 was considered as statistically significant.

### Qualitative analysis

A qualitative study was conducted among field-relevant stakeholders after the pilot period. In total, eleven participants agreed to be interviewed and were included through purposive sampling. This resulted in participants with extensive experience related to acute care for IPCBs in their profession or area of work. The included participants consisted of two police officers (‘officers on duty’), one 112 ambulance (national emergency call line) nurse, one ER nurse, one ER physician, three consultation–liaison–psychiatry physicians, one addiction care physician, one regional mental health crisis service psychiatrist and one employee from the municipal social service. A theoretical framework for conducting a semi-structured interview was constructed using literature-based implementation in healthcare studies [32, 33]. An independent researcher (FtD), who was not involved in the clinical care of IPCBs, performed the interviews. Participants were free to answer the questions in their own words. The collected data were analyzed, after transcription, through thematic content analysis with ATLAS.ti version 8.2.4 (Scientific Software Development GmbH, Berlin, DE). The coding method was established by coding two interviews independently by two researchers (SV and JvW). One researcher (SV) continued coding the remainder of the interviews. The results were coded both inductively and deductively until they could be merged into general themes. Two researchers then agreed upon the general themes (SV and JvW).

## Results

### Included subjects

A total of 142 IPCBs were included during the study period. The ER physician could include IPCBs if case description in the referral met the inclusion criteria. The regional 112-ambulance service referred most IPCBs (*N* = 95; 67%). In the case of 25 (18%) IPCBs, police officers were involved at first moment of the incident and 11 (8%) IPCBs were referred directly by the officer on duty from Arnhem police station. The ER physician referred 56 (39%) IPCBs for consultation–liaison–psychiatry physician consultation. After broad biopsychosocial assessment, 25 (18%) IPCBs were consecutively admitted to the complexity intervention unit. In the subgroup of IPCBs requiring police involvement at the first moment of the incident (*N* = 25), significantly more IPCBs were assessed by the consultation–liaison–psychiatry physician (*N* = 16; 64%; *P* < 0.01); this includes the smaller subgroup, which was referred directly by the officer on duty (*P* < 0.01).

### Process 1: demographics and patient routing

Table 1 shows the demographics and clinical features of the total study population (*N* = 142). Most IPCBs were male (*N* = 100; 70%); the average age of all IPCBs was 36.8 years (± 14.7 SD). Nine IPCBs (6%) were admitted multiple times to the ER during the 3-month pilot period: five of these were admitted to the complexity intervention unit. The mean age was significantly higher when IPCBs were assessed by the consultation–liaison–psychiatry physician (*N* = 56), were admitted to the complexity intervention unit (*N* = 25) or when police involvement was required (*N* = 16; *P* < 0.001, *P* < 0.001, and *P* = 0.007 respectively). Of all IPCBs, 25 (18%) were consecutively admitted to the complexity intervention unit; admission was compulsory for only six IPCBs (4%) and the police were involved in only three of these cases.

**Table 1:** Demographics study population

	Total study population	Assessment by consultation liaison psychiatry physician	Admitted to complexity intervention unit	Police officers required at first moment of incident
	*N* = 142	*N* = 56	*N* = 25	*N* = 25
Sex				
Male	100 (70%)	41 (73%)	21 (84%)	21 (84%)
Age (years)				
Mean ± standard deviation	36.82 (± 14.7)	44.9 (± 13.5)*** +	45.6 (± 12.7) *** +	44.2 (± 14.9)** +
Substance-related disorders				
Alcohol intoxication	78 (54%)	21 (38%) **-	10 (40%)	12 (48%)
Alcohol dependency	27 (19%)	21 (38%)*** +	13 (52%)*** +	4 (16%)
GHB intoxication	15 (11%)	1 (2%)*-	0 (0%)	2 (8%)
Amphetamine intoxication	10 (7%)	1 (2%)	1 (4%)	2 (8%)
Cannabis dependency	10 (7%)	4 (7%)	2 (8%)	2 (8%)
Cocaine intoxication	9 (6%)	3 (5%)	1 (4%)	1 (4%)
Alcohol withdrawal	7 (5%)	7 (13%)** +	4 (16%)* +	0 (0%)
Other substance abuse	32 (23%)	21 (38%)** +	7 (28%)	9 (36%)
Psychiatric disturbances				
Psychiatric disturbance present	65 (46%)	25 (45%)	14 (56%)	11 (44%)
Suicidal behavior	18 (13%)	16 (29%)*** +	7 (28%)* +	3 (12%)
Personality disorder	17 (12%)	13 (23%)*** +	8 (32%)** +	5 (20%)
Psychotic state	12 (8%)	10 (18%)** +	3 (12%)	5 (20%)
Cognitive impairment	5 (4%)	5 (9%)** +	5 (20%)*** +	1 (4%)
Delirium	9 (6%)	6 (11%)	4 (16%)	3 (12%)
Autism	5 (4%)	3 (5%)	2 (8%)	0 (0%)
Post-Traumatic Stress Disorder	5 (4%)	3 (5%)	1 (4%)	1 (4%)
Other psychiatric disorders	20 (14%)	14 (25%)** +	6 (24%)	5 (20%)
Somatic disturbances present	48 (34%)	32 (57%)*** +	19 (76%)*** +	8 (32%)
Current treatment in mental healthcare	#	33 (59%)	12 (48%)	12 (48%)
History of treatment in mental healthcare	#	49 (88%)	21 (84%)	14 (56%)
Compulsory admission	6 (4%)	5 (9%)* +	3 (12%)	3 (12%)
Re-visit ER with similar problem	9 (6%)	6 (11%)	5 (20%)** +	2 (8%)

Variables + are larger in group shown than in rest group (e.g., age is significantly higher in persons receiving extensive broad psychiatric, somatic and social assessment than persons without assessment); Variables—are smaller in group shown than in rest group (e.g., alcohol intoxication was less common in persons with an extensive broad psychiatric, somatic, and social assessment than without assessment)

***P < .001; **P < .01; *P < .05

^#^ These data were not collected by the ER physician and can only be determined in the subgroup consulted by a consultation–liaison–psychiatry physician

### Process 2: biopsychosocial examination and treatment

By far, alcohol-related problems were mostly seen in IPCBs at the ER [in total 73%; intoxication with alcohol (54%; *N* = 78) and alcohol dependency (19%; *N* = 27)]. These were also the most frequent problems at complexity intervention unit admission. Both ‘alcohol dependency’ and ‘withdrawal’, but not ‘alcohol intoxication’, appeared to be predictors for admission to the complexity intervention unit [*P* < 0.001, *P* = 0.015 and *P* = 0.125, respectively]. Almost half of all IPCBs (46%; *N* = 65) had a comorbid psychiatric problem. Suicidal behavior (13%; *N* = 18) and personality disorder related crisis (12%; *N* = 17) were most prevalent. In addition, other severe psychiatric disturbances were registered; notably, psychotic state (8%; *N* = 12), delirium (6%; *N* = 9), and mania (3%; *N* = 4). In the subgroup of IPCBs who were admitted to the complexity intervention unit, significantly more patients were registered with acute problems regarding a personality disorder (32%; *N* = 8), suicidal behavior (28%; *N* = 7), cognitive impairment (20%; *N* = 5) and delirium (16%; *N* = 4). Most of IPCBs who were examined by the consultation–liaison–psychiatry physician showed a history of former treatment in the mental healthcare system (88%; *N* = 45) and more than half received mental healthcare at the time (58%; *n* = 30).

Comorbid physical conditions were diagnosed in one third of the IPCBs (34%; *N* = 48) and many different somatic disorders were found; e.g., active rheumatoid arthritis, alcoholic hepatitis, injuries, and severe dehydration. The subgroup of IPCBs who were admitted to the complexity intervention unit showed significantly more comorbid physical conditions (76%; *N* = 19; *P* =  < 0.001). General functioning at complexity intervention unit admission was poor (GAF score of 24.6 ± 13.5 SD, out of a maximum score of 100 points).

The subgroup of IPCBs requiring police involvement showed no differences when compared with other subgroups, except that significantly more frequent use of coercion was needed at the ER (20%; *N* = 5; *P* < 0.01). By far, most (82%; *N* = 9) IPCBs who were referred by the officer on duty were not undergoing psychiatric treatment.

### INTERMED and HoNOS scores

**Fig. 1:**
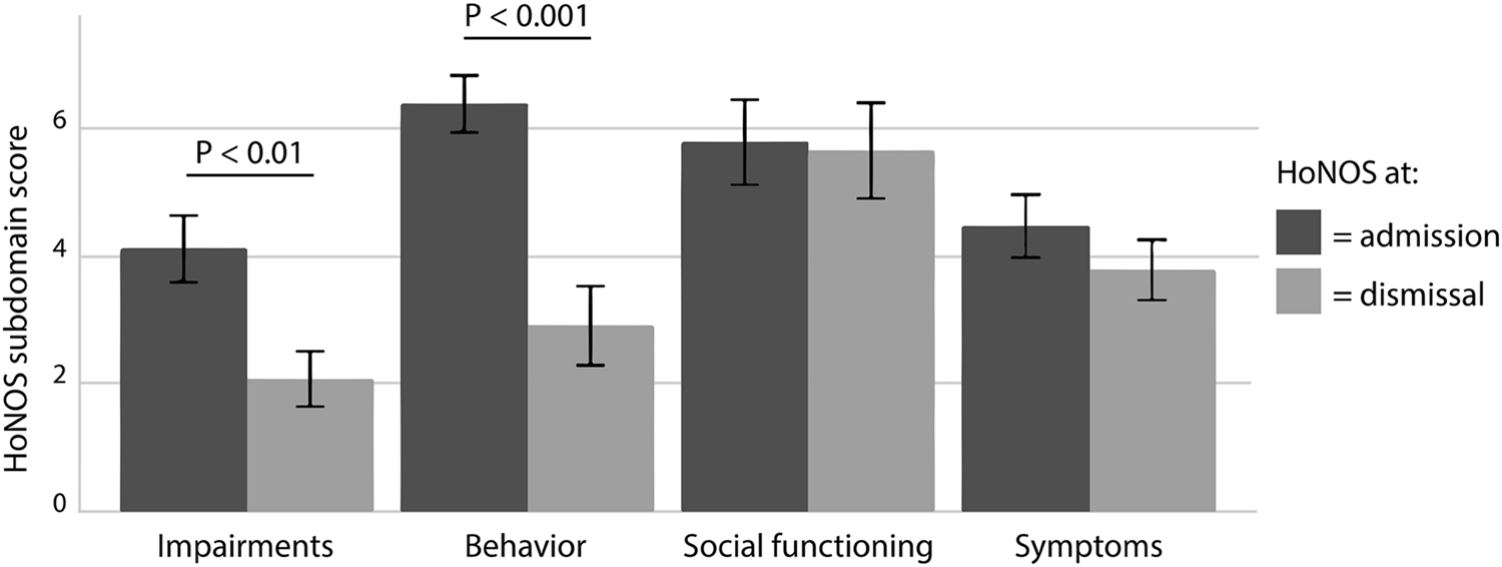
Health of the Nation Outcome Scale (HoNOS) subdomain-scores, at admission and discharge from the Complexity Intervention Unit

In the subgroup also examined by the consultation–liaison–psychiatry physician, an average INTERMED score of 27.8 ± 10.0 SD points was recorded (see Table 2). The subgroup of IPCBs requiring police involvement showed the highest average INTERMED score (30.8 ± 11.0 SD points). Both mean scores correspond to complex patients (INTERMED score > 19). In the subgroup that was consecutively admitted to the complexity intervention unit, a significantly worse prognosis on the INTERMED subdomain of ‘healthcare’ was observed (*P* < 0.05) as compared to those who were not admitted. In the subgroup of IPCBs requiring police involvement, significantly higher scores were shown on the INTERMED subdomain of ‘history psychologic’ (4.5 ± 1.7SD; *P* < 0.05), indicating additional vulnerabilities prior to admission. Using the HoNOS (see Table 3), IPCBs scored very high at complexity intervention unit admission (average HoNOS score was 20.8 ± 6.9 SD points) as compared to a reference group of regular admitted mental healthcare patients (average HoNOS score of 16.1 ± 7.3 SD points [28]). During their stay at the complexity intervention unit, the average total HoNOS score of the IPCBs diminished significantly to 13.4 ± 8.6 SD points at dismissal (*P* < 0.001); likewise, the mean subdomain scores also declined (Fig. 1).

**Table 2:** Mean INTERMED scores ± standard deviation. Differences analyzed through the Wilcox rank sum test

	Assessment by consultation-liaison psychiatry physician	Admitted to complexity intervention unit	Police officers required at first moment of incident
	*N* = 56 (100%)	*N* = 25 (45%)	*N* = 16 (29%)
Biologic			
History	2.39 (± 1.6)	2.52 (± 1.7)	2.31 (± 1.7)
Current state	2.86 (± 1.6)	3.20 (± 1.7)	3.00 (± 2.0)
Prognosis	0.71 (± 1.0)	0.80 (± 1.1)	0.69 (± 1.0)
Psychologic			
History	4.04 (± 1.37)	4.16 (± 1.4)	4.5 (± 1.67) * +
Current state	3.46 (± 1.39)	3.44 (± 1.56)	4.00 (± 1.37)
Prognosis	1.79 (± 0.65)	1.72 (± 0.68)	2.00 (± 0.73)
Social			
History	3.71 (± 1.91)	4.04 (± 1.51)	4.38 (± 1.50)
Current state	2.88 (± 1.74)	3.40 (± 1.73)	3.19 (± 1.91)
Prognosis	0.93 (± 0.85)	1.16 (± 0.85)	1.12 (± 0.96)
Healthcare			
History	1.82 (± 1.60)	1.96 (± 1.9)	2.06 (± 1.69)
Current state	1.73 (± 1.07)	1.80 (± 1.26)	1.81 (± 1.33)
Prognosis	1.46 (± 0.87)	1.80 (± 0.82) * +	1.69 (± 0.95)
Total score	27.79 (± 9.97)	30.00 (± 11.49)	30.75 (± 10.98)

^***^P < .001; **P < .01; *P < .05

**Table 3:** Mean Health of the Nation Outcome Scale (HoNOS)-scores ± standard deviation, at admission to Complexity Intervention Unit. Differences analyzed through the Wilcox rank sum test

	Admitted to complexity intervention unit	Police officers required at first moment of incident
	*N* = 24 (96%)	*N* = 6 (25%)
HoNOS subdomains		
Behavior	6.32 (± 2.3)	7.17 (± 0.75)
Impairment	3.96 (± 2.6)	2.50 (± 3.21)
Symptoms	4.52 (± 2.50)	3.67 (± 1.37)
Social	5.8 (± 3.35)	4.83 (± 2.99)
Total score	20.6 (± 6.9)	18.17 (± 6.01)

^***^P < .001; **P < .01; *P < .05

### Process 3: follow-up care

A mortality rate of 1.4% during the study period was found (suicide: *N* = 1; acute liver failure: *N* = 1). On average, IPCBs stayed at the ER for 2.8 ± 1.8 SD hours. The subgroup of IPCBs who were assessed using the INTERMED stayed on average half an hour longer (3.2 ± 1.9 SD versus 2.8 h) than all ER patients in that period (including patients that were referred to other medical departments). Of IPCBs seen by the ER physician, 78 (55%) were discharged home; referred to their general practitioner for any additional help and were lost in follow-up. After broader biopsychosocial assessment at the ER, 26 (19%) were referred to outpatient mental health facilities, including addiction care. This was only possible for IPCBs with a relatively mild, sole psychiatric or social problem that had no medical indication for admission to a hospital. This was also the case if an IPCB preferred the follow-up with their own mental health professional.

Twenty-five IPCBs (18%) who were admitted to the complexity intervention unit stayed an average of 37 ± 30.4 SD hours; ten IPCBs (40% of the complexity intervention unit-admitted IPCBs) stayed more than 140 h, because the applicable follow-up care appeared very complicated to arrange. After short-term complexity intervention unit admission, 14 (56%) IPCBs needed prolonged psychiatric admission and nine IPCBs (36%) were referred to outpatient mental healthcare facilities, including addiction care. Only a very small subgroup of IPCBs required exclusively social services without further physical or mental healthcare (2%; *N* = 3). Two IPCBs (2%) could not be referred to appropriate follow-up care: one person (1%) had to return to a facility of the criminal justice department, one person (1%) denied more help. On average, the time interval until start of the appropriate follow-up care after the initial assessment was 7.3 ± 10.2 SD days. However, available data points for this analysis were limited.

### Qualitative analysis

Two main themes emerged from the qualitative analyses: (1) general satisfaction of designating the ER for IPCBs and (2) quality and efficiency of referral between different care providers (see Table 4). The most reported satisfactory comments were: “At the ER, IPCBs are in the adequate place to get help” (reported 18 times); “During the pilot period, there was a better flow-through in the healthcare system” (reported 15 times); and “During the pilot period, there was better cooperation between referral first responding parties” (reported 12 times). Some participants “did not notice a difference in their daily routine” (reported four times). There were also participants who “did not notice a difference in the flow-through of the healthcare system” (reported four times). Two participants (ER physician and ER nurse) reported both these comments most frequently (reported 3 times and 3 times, respectively). According to the interviewed participants, there were still improvements to make: they mentioned “noticeable aversion to psychiatric patients in the ER” (reported 14 times; consultation–liaison–psychiatry physicians reported this most frequently at 9 times), “Referral to follow-up care could have been faster” (reported 9 times), and “There is still uncertainty about what is the best option for referral to follow-up care” (reported 8 times). In general, the interviewed participants were positive about the idea of designating the ER for physical and mental examination of IPCBs. The interviewed participants working *in* the ER were less positive, however, since they did not notice any difference during the pilot period.

**Table 4:** Report of how many times a statement (as quoted) has been mentioned during an interview with a participant

	Admission				Assess-ment	Referral			
		Ambu-lance	Police	ER	Psychia-try Ward	Mental Health Care Facility	Depen-dency Health- care	Munici-pality	Total times reported
Improvements made during pilot									
Number of participants per referral party		1	2	2	3	1	1	1	
In ER: IPCB in adequate place for help		2	9	0	4	2	1	0	18
Smoother flow-through the healthcare system		1	5	0	7	0	2	0	15
Better cooperation between referral parties		1	2	0	4	0	5	0	12
No difference noticed									
No difference in daily routine		0	1	3	0	0	0	0	4
No difference in flow-through the healthcare system		0	0	3	1	0	0	0	4
Still to improve									
Noticeable aversion towards psychiatric patients in Emergency Department		1	0	4	9	0	0	0	14
Referral to follow-up care could have been faster		0	4	1	3	0	1	0	9
Still uncertainty for best option referral for follow-up care		0	4	1	3	0	0	0	8

This table only contains an overview of the most reported statements

## Discussion

In this pilot study, a broad biopsychosocial assessment of intoxicated persons showing challenging behavior at the ER elucidated several medical diagnoses in many cases, for which essential treatments and appropriate follow-up care were arranged. All within a reasonable time interval. Almost half of IPCBs showed serious psychiatric disturbances and one third suffered from significant physical conditions. All these medical conditions warranted acute medical attention and/or treatment to reduce the risks of serious consequences. Additionally, high complexity scores (INTERMED and HoNOS) as well as low general functioning scores (GAF) indicate that IPCBs suffer from severe and complex biopsychosocial disturbances and disabilities. Owing to this complexity, an interdisciplinary approach in the emergency healthcare for IPCBs was essential. Therefore, the ER appeared to be the best place to receive and assess these patients.

### Treatment optimism

The findings of our pilot study were very hopeful. During their stay at the hospital, the average HoNOS score of IPCBs declined significantly. Most likely due to improvements of the HoNOS subdomains ‘behavior’ (e.g., aggression, substance abuse) and ‘disability’ (e.g., cognitive problems, somatic disorders) at discharge from the complexity intervention unit. Challenging behavior decreased or even disappeared in less than two days. Although this care was executed within the separated Dutch healthcare systems, the biopsychosocial approach at the ER proved not to be powerless at all. It showed that it leads to more appropriate care for IPCBs. Furthermore, almost all (96%) IPCBs accepted help voluntarily and the duration of their stay at the ER was not significantly different from other ER patients. According to this pilot study, the stigmatizing assumptions of caregivers that most IPCBs will reject help, will be unmanageable, will only suffer from social problems or crises, and will demand much more time from the busy ER staff, seem—therefore—incorrect [13].

### Merging physical, mental healthcare and social services

To improve the emergency healthcare for IPCBs both physical and mental healthcare institutions should be merged. The qualitative data showed a broad support amongst all field-relevant stakeholders for designating the ER to receive and assess IPCBs with emergency healthcare needs. In our interviews, it was also mentioned that the general attitude towards IPCBs should be improved, especially at the ER. The well-known negative attitude towards psychiatric patients in the ER may increase self-stigmatization among IPCBs, leading to a further decrease in help-seeking behavior [34]. Transforming this negative attitude into a more positive one, can add substantial value for patients, caregivers, police officers, and ER staff. A more biopsychosocial approach at the ER may decrease the stigmatization of IPCB, but may also improve job satisfaction for all involved professionals. Specific education, training and exposure of all healthcare professionals to ‘confused persons’ on a regular basis may help instigate this urgently needed transformation. Furthermore, social services could be involved more often, to assess social problems since a short-term admission does not diminish HoNOS scores related to social problems. This might be due to the fact that social problems are often long-term and require more time to resolve (e.g., homelessness, debts, relationship problems) as well as the fact that patients have comorbid medical problems that need attention first.

### Fruitful cooperation with police

As expected, the police officers were often the first responders to provide help to IPCBs. The police officers were shown to refer IPCBs adequately to the ER. IPCBs requiring police involvement showed the highest average total INTERMED score, as compared to the other IPCBs, indicating that they suffered from the most complex problems. Interestingly, 82% of IPCBs directly referred by the officer on duty had no current mental healthcare provider, although (after assessment) 44% of IPCBs suffered psychiatric disturbance and 32% a physical illness. Both these findings point at the alarming situation that these IPCBs—probably—would not have been given appropriate and necessary assessment, treatment, and/or follow-up care in the period before our pilot study. Physical and mental healthcare professionals may, therefore, value the police officers’ involvement more since they deliver these patients into the healthcare systems.

## Limitations

Some critical remarks should be considered when interpreting the results of this study. During this pilot period, a more comprehensive biopsychosocial examination and the option for a short-term admission to our complexity intervention unit seem to add much-needed value to the care for IPCBs. However, the added values for IPCBs were not examined in a randomized controlled manner; comparing the regular ER practice with the pilot situation of ER designation for integrated biopsychosocial care.

Another limitation was the relatively small number of included IPCBs, due to the naturalistic nature of this study. Furthermore, due to the explorative nature of the study, no adjustments were made to account for multiple statistical comparisons. Although this may have decreased the reliability of the results, the reliability of the main findings on comorbidity in IPCBs is suggested by its consistency with previous research [9].

In advance, the subgroup of IPCBs directly referred by the officer on duty was expected to be much larger. In the qualitative interviews, the officers on duty stated that “they did not want to abuse the option to refer [IPCBs] to the ER.” They stated that, in hindsight, more IPCBs could have been referred by the police because only IPCBs from the city of Arnhem were allowed to be referred. The hospital, however, has a much larger catchment area with more surrounding police departments that would very much like to refer IPCBs.

Another remark was that the improvement of the HoNOS scores (e.g., impairment, behavior) during the stay at the complexity intervention unit may not have been the exclusive effect of the treatments given. IPCBs sobered up spontaneously in the given time interval and this also may have had a significant effect on the challenging behavior. Generally speaking, however, this examined population, which was nsot directly affected by an acute intoxication with alcohol and/or drugs, still scored high on many other HoNOS subscores. Finally, the majority of the IPCBs discharged home were referred to their general practitioner for any additional help and were subsequently lost in follow-up.

## Conclusion

This pilot aimed to substantiate the physical, psychiatric, and social health needs of IPCBs who visited the emergency room (ER) during a 3-month period. The pilot showed that integrated healthcare is needed for IPCBs at the ER because these patients often suffer from substantial biopsychosocial conditions, disturbances, and problems. A voluntarily accepted, more comprehensive biopsychosocial assessment at the ER by consultation–liaison psychiatric staff, sometimes followed by integrated physical and mental health care at a specialized complexity intervention unit, may significantly improve short-term outcomes. All within a reasonable time interval. The police appear to be a trustworthy referrer to the ER and will deliver patients in great need of help. All field-relevant stakeholders strongly supported the implementation of short-term biopsychosocial assessment and intervention programs for IPCBs at both the ER and complexity intervention unit of the hospital. This approach may help to merge the currently ineffectively separated healthcare systems. This will not only greatly improve emergency healthcare, but also will reduce the stigmatization of these people in need.

## Data Availability

Data cannot be shared publicly because of Dutch privacy law regarding patient data. Data are available from Rijnstate Hospital Psychiatry Department (contact via corresponding author) for researchers who meet the criteria for access to confidential data.
